# Pulmonary Embolism due to Inferior Vena Cava Compression by a Retroperitoneal Hematoma after Endovascular Repair of a Ruptured Abdominal Aortic Aneurysm

**DOI:** 10.1155/2017/8172549

**Published:** 2017-05-17

**Authors:** Kota Shukuzawa, Naoki Toya, Yasutake Momokawa, Soichiro Fukushima, Tadashi Akiba, Takao Ohki

**Affiliations:** ^1^Department of Vascular Surgery, The Jikei University School of Medicine, Tokyo 108-8461, Japan; ^2^Division of Vascular Surgery, Department of Surgery, The Jikei University Kashiwa Hospital, Chiba 277-8567, Japan; ^3^Department of Surgery, The Jikei University Kashiwa Hospital, Chiba 277-8567, Japan

## Abstract

We report a case of a patient with a residual hematoma compressing the inferior vena cava after endovascular aneurysm repair (EVAR), which led to a pulmonary embolism (PE). A 65-year-old man underwent emergent EVAR for a ruptured aortic aneurysm in the right retroperitoneal region. He developed sudden chest pain at midnight of the fifth day after EVAR, and computed tomography demonstrated a massive PE. He subsequently went into cardiopulmonary arrest. This case suggested that abdominal complications due to a residual hematoma, including deep vein thrombosis and PE, should be considered in addition to compartment syndrome.

## 1. Introduction

The results of some reports support the benefits of endovascular aneurysm repair (EVAR) compared with those of open surgical repair (OSR) for managing a ruptured abdominal aortic aneurysm (rAAA) [1, 2]. EVAR is less invasive in patients with favorable anatomy. However, it is not possible to remove a hematoma by EVAR. We present a case of a patient with a pulmonary embolism (PE) due to compression of the inferior vena cava (IVC) by a residual hematoma after EVAR for an rAAA. Consent was obtained from the patient's family to publish this case report.

## 2. Case Presentation

**Figure 1:**
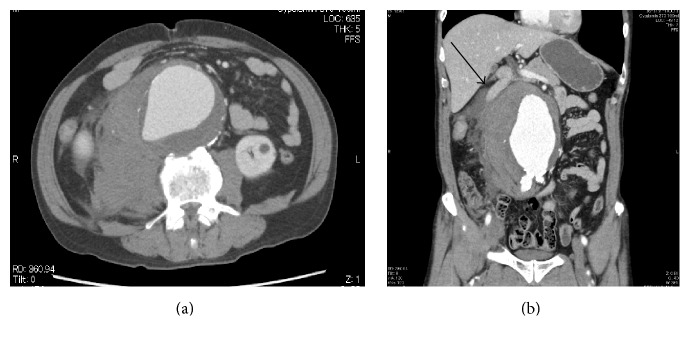
(a) Enhanced computed tomography revealing a ruptured aortic aneurysm measuring 7.9 × 7.6 cm in diameter in the right retroperitoneal region. (b) The inferior vena cava is compressed by a hematoma (black arrow).

**Figure 2:**
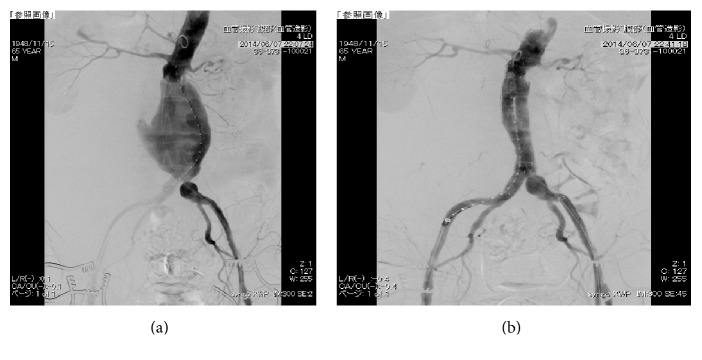
Angiogram before (a) and after (b) endovascular aneurysm repair.

A 65-year-old man was admitted to our emergency department because of sudden back pain and hypotension (systolic blood pressure, 80 mmHg). Unmedicated hypertension was noted at the time of admission. He had no signs of deep vein thrombosis (DVT) in his legs, such as obvious edema. Enhanced computed tomography (CT) demonstrated the presence of an rAAA, measuring 7.9 × 7.6 cm in diameter, in the right retroperitoneal region (Figure 1). The patient underwent emergent EVAR using the Excluder stent graft (W. L. Gore & Associates, Inc., Flagstaff, Arizona) (Figure 2). Initially, the postoperative course was uneventful without an elevation in intravesical pressure. As the initial postoperative CT revealed no evidence of an endoleak at 3 days after EVAR, the patient was extubated and oral intake was started. At midnight of the fifth day after EVAR, he complained of sudden chest pain on walking. He subsequently went into cardiopulmonary arrest.

**Figure 3:**
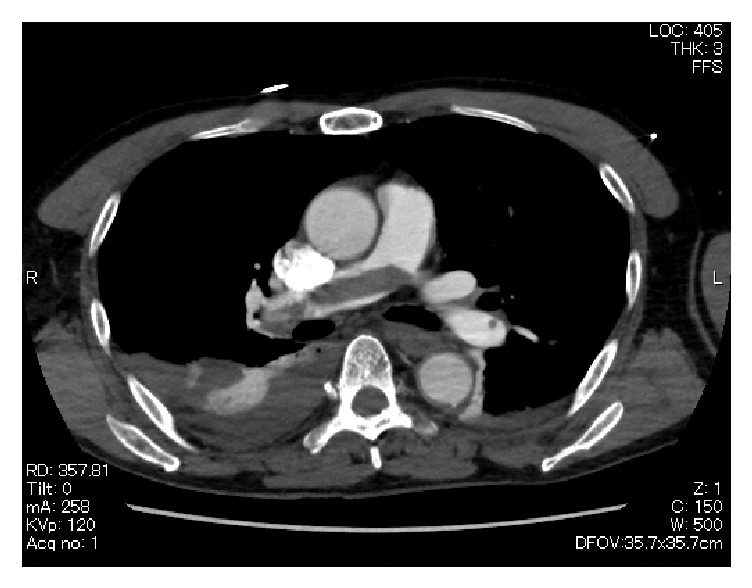
Enhanced computed tomography revealing a pulmonary embolism (white arrow).

Echocardiography revealed right ventricular dysfunction, and CT revealed a massive pulmonary embolism (Figure 3). Urokinase (5,000 IU/kg) was intravenously administered. Return of spontaneous circulation was achieved after successful cardiopulmonary resuscitation and intravenous vasoconstrictor injection. However, hypoxic encephalopathy unfortunately remained. At 90 days after EVAR, he was transferred to a care hospital with a respirator.

## 3. Discussion

The use of EVAR for rAAA remains limited by the risk of abdominal compartment syndrome and residual hematomas. There are some published reports of large aneurysms compressing the IVC and inducing venous thromboembolisms [3, 4]. A retroperitoneal hematoma due to an rAAA rarely causes PE [5]. To our knowledge, this is the first instructive report on a residual hematoma compressing the IVC after EVAR which led to PE.

This case highlights the importance of complications due to a residual hematoma after EVAR for an rAAA. de Maistre et al. [6] reported that DVT may be more frequent following OSR (10.2%) than following EVAR (5.3%) in the treatment of an abdominal aortic aneurysm (AAA). The three risk factors associated with thrombosis are widely known such as Virchow's triad, which consists of vessel wall damage, blood flow stasis, and hypercoagulability. In cases of an rAAA, coagulability worsens because of massive hemorrhage and the presence of hematomas within the IVC or iliac vein, directly compressing the blood flow. Therefore, DVT might be more frequent in an rAAA than in an unruptured AAA. The use of OSR allows the removal of hematomas during surgery, whereas EVAR prevents such removal. This is a major disadvantage of EVAR.

In the present case, the initial physical examination revealed no signs of a venous thromboembolism. After EVAR, slight edema was observed which affected the entire body and not just the legs. We considered this edema to be due to the large volume of transfusion. Furthermore, we did not use an anticoagulant to prevent DVT because of the risk of postoperative hemorrhage from an endoleak. Although CT revealed no obvious sign of a venous thromboembolism at 3 days after EVAR, the main indication for CT at this time was to look for a potential endoleak. Therefore, CT of the leg was not performed. Echography of the inguinal region and legs may have been useful.

In conclusion, after EVAR for rAAAs, clinicians should consider abdominal complications due to a residual hematoma, including DVT and a PE, in addition to compartment syndrome. Although EVAR is less invasive in patients with favorable anatomy, it is limited by an inability to remove hematomas in cases of rAAAs. Residual hematomas, particularly when located on the right side, may be a cause of DVT. Therefore, strict surveillance is required after EVAR for rAAAs. Furthermore, if necessary, the placement of a prophylactic IVC filter or secondary open conversion to remove the hematoma should be considered.
